# Diagnosis and treatment of disseminated intravascular coagulation (DIC) according to four DIC guidelines

**DOI:** 10.1186/2052-0492-2-15

**Published:** 2014-02-20

**Authors:** Hideo Wada, Takeshi Matsumoto, Yoshiki Yamashita

**Affiliations:** Department of Molecular and Laboratory Medicine, Mie University School of Medicine, 2-174 Edobashi, Tsu, Mie, 514-8507 Japan; Department of Blood Transfusion, Mie University School of Medicine, 2-174 Edobashi, Tsu, Mie, 514-8507 Japan; Department of Hematology and Oncology, Mie University School of Medicine, 2-174 Edobashi, Tsu, Mie, 514-8507 Japan

**Keywords:** Disseminated intravascular coagulation (DIC), Bleeding type, Organ failure type, Massive bleeding type, Non-symptomatic type, Guidelines

## Abstract

Disseminated intravascular coagulation (DIC) is categorized into bleeding, organ failure, massive bleeding, and non-symptomatic types according to the sum of vectors for hypercoagulation and hyperfibrinolysis. The British Committee for Standards in Haematology, Japanese Society of Thrombosis and Hemostasis, and the Italian Society for Thrombosis and Haemostasis published separate guidelines for DIC; however, there are several differences between these three sets of guidelines. Therefore, the International Society of Thrombosis and Haemostasis (ISTH) recently harmonized these differences and published the guidance of diagnosis and treatment for DIC. There are three different diagnostic criteria according to the Japanese Ministry Health, Labour and Welfare, ISTH, and Japanese Association of Acute Medicine. The first and second criteria can be used to diagnose the bleeding or massive bleeding types of DIC, while the third criteria cover organ failure and the massive bleeding type of DIC. Treatment of underlying conditions is recommended in three types of DIC, with the exception of massive bleeding. Blood transfusions are recommended in patients with the bleeding and massive bleeding types of DIC. Meanwhile, treatment with heparin is recommended in those with the non-symptomatic type of DIC. The administration of synthetic protease inhibitors and antifibrinolytic therapy is recommended in patients with the bleeding and massive bleeding types of DIC. Furthermore, the administration of natural protease inhibitors is recommended in patients with the organ failure type of DIC, while antifibrinolytic treatment is not. The diagnosis and treatment of DIC should be carried out in accordance with the type of DIC.

## Introduction

Disseminated intravascular coagulation (DIC) is a syndrome characterized by the systemic activation of blood coagulation, which generates intravascular thrombin and fibrin, resulting in the thrombosis of small- to medium-sized vessels and ultimately organ dysfunction and severe bleeding [1, 2]. DIC may result as a complication of infection, solid cancers, hematological malignancies, obstetric diseases, trauma, aneurysms, and liver diseases, etc., each of which presents characteristic features related to the underlying disorder. The diagnosis and treatment of DIC must therefore consider these underlying etiological features. The type of DIC is related to the underlying disorder. Three guidelines for diagnosis and treatment of DIC [3–5] have been published in the literature by the British Committee for Standards in Haematology (BCSH), Japanese Society of Thrombosis and Hemostasis (JSTH), and Italian Society for Thrombosis and Haemostasis (SISET). Although these three guidelines are broadly similar, there are variations in several recommendations regarding DIC treatment. Therefore, the subcommittee for DIC of the Scientific and Standardization Committee (SSC)/International Society of Thrombosis and Haemostasis (ISTH) harmonized these three guidelines in a report entitled, *Guidance for the diagnosis and treatment of DIC from harmonization of the recommendations from three guidelines*[6] (Table 1). The present review describes several recommendations for the diagnosis and treatment of DIC related to the type of DIC.

**Table 1:** **Differences in recommendations among three guidelines from BCSH, JSTH, and SISET and harmonized ISTH/SSC guidance**

	BCSH	JSTH	SISET	ISTH/SSC
Scoring system for DIC	R; grade C	R^a^	R; grade C	R; high quality
Single test analysis for DIC	NR	NR^a^	NR; grade D	R high quality
Treatment of underlying disease	R; grade C	R; consensus	R; cornerstone	R; moderate quality
Platelet concentration	R; grade C	R; consensus	R; grade D	R; low quality
FFP	R; grade C	R; consensus	R; grade D	R; low quality
Fibrinogen, cryoprecipitate	R; grade C	Disregard	R; grade D	R; low quality
FVIIa	Disregard	Disregard	NR; grade D	NM
UFH (treatment)	R; grade C	R; level C	NR; grade D	R; low quality
UFH (prophylaxis for VTE)	R; grade A	Disregard	R	R; high quality
LMWH	Disregard	R; level B2	R; grade D	Preferred to UFH
Heparin sulfate	Disregard	R; level C		NM
Synthetic protease	Disregard	R; level B2	NR; grade D	NM
rhAPC	R; grade A	Disregard	R; grade D	Need for further Ed from RCT
AT	NR; grade A	R; B1	NR; grade D	Need for further Ed from RCT
rhTM	Disregard	Disregard	NR; grade B	Need for further Ed from RCT
Antifibrinolytic agents	R; grade C	NR; level D		R; low quality
Plasma exchange	Disregard	Disregard	NR; grade D	NM

R, recommendation; NR, not recommendation; R^a^, suggestive recommendation; NM, not mention; Ed, evidence; FFP, fresh frozen plasma; PCC, FVIIa, activated coagulation factor VII; UFH, unfractionated heparin; LMWH, low molecular weight heparin; rh, recombinant human; APC, activated protein C; AT, antithrombin; TM, thrombomodulin; RCT, randomized control trial.

## Review

### Pathophysiology of DIC

**Figure 1:**
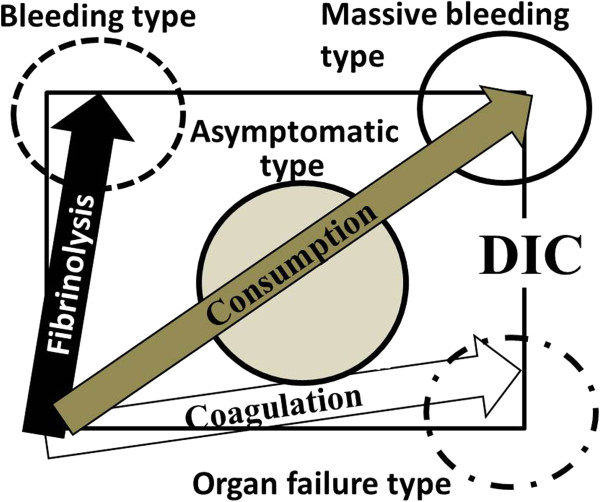
**Bleeding, organ failubre, massive bleeding, and non-symptomatic types of DIC.**

Abnormalities of the hemostatic system in patients with DIC result from the sum of vectors for hypercoagulation and hyperfibrinolysis (Figure 1). When the vector for hyperfibrinolysis is remarkable and dominant, bleeding is the primary symptom; this type is called the bleeding type or hyperfibrinolysis predominance type of DIC. This form of DIC is often seen in patients with leukemia, such as acute promyelocytic leukemia (APL), obstetric diseases, or aortic aneurysms [2, 7]. On the other hand, when the vector for hypercoagulation is remarkable and dominant, organ failure is the main symptom; this type of DIC is called the organ failure type, hypercoagulation predominance type or hypofibrinolysis type of DIC. This form of DIC is often observed in patients with infection, particularly sepsis. An increase in the level of plasminogen activator inhibitor I (PAI-I) induced by markedly increased levels of cytokines [8, 9] and lipopolysaccharide (LPS) [2, 7] in the blood has been reported to a cause of hypofibrinolysis. Moreover, neutrophil extracellular traps (NETs) [10], which release DNA with histone, neutrophil elastase, and cathepsin G in order to trap and kill pathogens, are present in patients with sepsis. Histones promote the apoptosis of vascular endothelial cells and platelet aggregation [11], while neutrophil elastase and cathepsin G decompose tissue factor pathway inhibitor (TFPI) in order to promote thrombus formation [12]. Moreover, high mobility group box 1 (HMGB-1) [13] is emitted from injured and dead cells in order to enhance the inflammatory reaction.

When both vectors for hypercoagulation and hyperfibrinolysis are remarkable and strong, major bleeding occurs, followed by death, if a sufficient amount of blood is not transfused; this type of DIC is called the massive bleeding or consumptive type of DIC. This form of DIC is observed in patients who exhibit major bleeding after major surgery or in those with obstetric diseases.

When both vectors are weak, there are almost no clinical symptoms, although abnormalities in clinical laboratory tests are observed; this type of DIC is called the non-symptomatic type of DIC or pre-DIC [14, 15]. In a retrospective study [15], the treatment of pre-DIC was reported to be effective. The diagnosis and treatment of the four types of DIC differ [3]. Furthermore, the diagnosis and treatment of DIC is complicated by the fact that the types of DIC may shift or change. Patients with DIC caused by sepsis (organ failure type), hematological malignancy, or obstetrics (bleeding type) can be successfully treated for DIC, whereas DIC associated with solid cancers may not respond to standard treatments [16]. As DIC associated with solid cancers differs from the above four types of DIC, it should be analyzed separately.

### Diagnosis of DIC

#### Scoring system

Various underlying clinical conditions can have an effect on the laboratory parameters that are usually obtained to diagnose DIC, such as global coagulation tests, the platelet count, prothrombin time (PT), and the fibrinogen, fibrinogen, and fibrin degradation products (FDPs). In order to facilitate the diagnostic process for detecting DIC, the use of a scoring system is recommended by each of the four different guidelines [3–6]. Three different diagnostic criteria incorporating similar global coagulation tests have been established by the ISTH/SSC [1], Japanese Ministry Health, Labour and Welfare (JMHLW) [17], and Japanese Association of Acute Medicine (JAAM) [18]. The JMHLW score is well correlated with the severity of DIC and can be used to predict the outcome of the disease [14]. The ISTH overt DIC score is useful and specific for diagnosing DIC due to infective and non-infective etiologies [13, 19]. The JAAM score is sensitive for detecting septic DIC and is correlated with the ISTH and JMHLW scores and disease outcome [13, 18]. A prospective study in Japan reported no significant differences in the odds ratio for predicting DIC outcomes among these three diagnostic criteria [20], suggesting that the identification of molecular hemostatic markers and changes of global coagulation tests is required in addition to the application of scoring systems. The use of a combination of tests repeated over time in patients with suspected DIC can be used to diagnose the disorder with reasonable certainty in most cases [21–23]. A template for a non-overt-DIC scoring system, including global coagulation tests, changes in global coagulation tests as well as hemostatic molecular markers, has been proposed [1, 24, 25].

The bleeding type of DIC can be easily diagnosed using the ISTH overt-DIC [1] and JMHLW [17] criteria, while the organ failure type of DIC is diagnosed according to the JAAM diagnostic criteria [18]. The massive bleeding (consumptive) type of DIC can be diagnosed using any of the three diagnostic criteria [1, 17, 18]; however, it is difficult to diagnose the non-symptomatic type of DIC using these criteria. The use of hemostatic molecular markers is required to diagnose the non-symptomatic type of DIC.

#### Laboratory tests

Global coagulation tests provide important evidence regarding the degree of coagulation factor activation and consumption. Although the PT is prolonged in approximately 50% of patients with DIC at some point during their clinical course [21], abnormalities are often observed in patients with liver disease or vitamin K deficiency. A reduction in the platelet count or clear downward trend in subsequent measurements is a sensitive sign of DIC [3], although this pattern is also observed in patients with bone marrow disorders. A reduced fibrinogen level is a valuable indicator regarding a diagnosis of DIC due to leukemia or obstetric diseases; however, it is not observed in most septic DIC patients [3]. Elevated fibrin-related markers (FRMs), such as FDP [26], D-dimer [27], or soluble fibrin (SF), reflect fibrin formation. SF [28] assays offer theoretical advantages in detecting DIC, more closely reflecting the effects of thrombin on fibrinogen, although the half-life is short. It is important to consider that many conditions, such as trauma, recent surgery, bleeding, or venous thromboembolism (VTE), are associated with elevated FRMs. Reductions in the levels of natural anticoagulants, such as antithrombin (AT) and protein C, are common in patients with DIC. Although measuring the AT activity is useful for achieving the full efficacy of heparin [29], this parameter cannot be quickly and easily measured in all hospitals. These activities are correlated with the liver function and/or concentration of albumin. A reduced ADAMTS13 (a disintegrin-like and metalloproteinase with thrombospondin type 1 motifs 13) activity and elevated soluble thrombomodulin (TM), PAI-I, and von Willebrand factor propeptide levels are often observed in patients with DIC and have been shown to have prognostic significance [30–32]. The biphasic waveform of the activated partial thromboplastin time (APTT) has been shown to be associated with DIC and appears to have a positive predictive value for the disease [33, 34]. Although many attractive markers for DIC have been reported, no single marker can be used to diagnose DIC alone (Table 2). Therefore, the above four guidelines [3–6] recommend that DIC could not be diagnosed according to the level of a single marker but rather based on the combination of laboratory markers. Among the four types of DIC, PT, fibrinogen, and platelets are important parameters for diagnosing the massive bleeding type of DIC, while fibrinogen, FDP, and plasmin-plasmin inhibitor complex (PPIC) are important for detecting the bleeding type of DIC. Meanwhile, platelets, PT, and AT are important for diagnosing the organ failure type of DICand hemostatic molecular markers, such as SF and the thrombin-AT complex, are important for diagnosing the non-symptomatic type of DIC.

**Table 2:** **Laboratory tests for DIC**

	Abnormality in DIC	Other cause for the abnormality	Adequate type of DIC
PT	Prolongation	Liver dysfunction, vitamin K deficiency	OF, BL, MB
FDP, D-dimer	Elevation	Venous thromboembolism, operation	BL, NS, OF
Fibrinogen	Reduction	Liver dysfunction	BL, MB
Platelet count	Reduction	Bone marrow disorders	OF, MB, BL, NS
AT/PC	Reduction	Liver dysfunction, capillary leak syndrome	OF
SF/TAT	Elevation	Venous thromboembolism, operation	OF, NS, BL, MS
TM	Elevation	Renal dysfunction, organ failure	OF
VWFpp, PAI-I	Elevation	Organ failure	OF
ADATMTS13	Reduction	Liver dysfunction, thrombotic microangiopathy	OF
APTT	Biphasic waveform	Infection	OF
PPIC	Elevation	Venous thromboembolism, operation	BL, MB

PT, prothrombin time; FDP, fibrinogen and fibrin degradation products; SF, soluble fibrin; AT, antithrombin; PC, protein C; TAT, thrombin AT complex; VWFpp, von Willebrand factor propeptide; PAI-I, plasminogen activator inhibitor-I; APTT, activated partial thromboplastin time; PPIC, plasmin-plasmin inhibitor complex; OF, organ failure type of DIC; BL, bleeding type of DIC; MB, massive bleeding type of DIC; NS, non-symptomatic type of DIC.

### Treatment of DIC

#### Treatment of the underlying disease

The cornerstone of DIC treatment is providing treatment for the underlying disorders, such as the administration of antibiotics or surgical drainage in patients with infectious diseases and anticancer drugs or surgery in patients with malignant diseases. All four guidelines [3–6] agree on this point, although there is no high-quality evidence for the efficacy of treating the underlying disorder in DIC patients. DIC spontaneously resolves in many cases when the underlying disorder is properly managed and improved. However, some cases require additional supportive treatment specifically aimed at abnormalities in the coagulation system. A randomized controlled trial (RCT) of the use of all-trans retinoic acid (ATRA) compared with conventional chemotherapy in patients with APL showed that the mortality rate was significantly lower in the ATRA group [35]. ATRA exerts differential effects on APL progression, as well as anticoagulant and antifibrinolytic effects [36]. Similarly, several RCTs of the treatment of sepsis [37–42] and DIC [43] have shown parallel improvements in coagulation derangement and DIC, although the data have not always been concordant. Treating the underlying disorder is first required in patients with bleeding, organ failure, and non-symptomatic types of DIC, while blood transfusions are needed in patients with the massive bleeding type of DIC (Table 3).

**Table 3:** **Treatment of DIC in four types of DIC**

Treatment	Non-symptomatic type	Organ failure type	Bleeding type	Massive bleeding type
Underlying conditions	R	R	R	
Blood transfusion			R	R
Heparin	R		NR	NR
Anti-Xa			NR	NR
Synthetic protease inhibitor			R	R
Natural protease inhibitor		R		NR
Antifibrinolytic treatment	NR	NR	R	R

R, recommended; NR, not recommended.

#### Blood transfusion

Markedly low levels of platelets and coagulation factors, particularly fibrinogen, may increase the risk of bleeding. The above four guidelines [3–6] recommended the administration of platelet concentrate (PC) and fresh frozen plasma (FFP) in DIC patients with active bleeding or those at high risk of bleeding requiring invasive procedures, without high-quality evidence. The threshold for transfusing platelets depends on the clinical state of the DIC patient. In general, PC is administered in DIC patients with active bleeding and a platelet count of ≦50 × 10^9^/l. A much lower threshold of 10 to 20 × 10^9^/l is adopted in non-bleeding patients who develop DIC after undergoing chemotherapy. PC may be administered at higher levels in patients perceived to be at high risk of bleeding based on other clinical or laboratory features [44]. The transfusion of PC or FFP is usually performed in patients with the massive bleeding or bleeding types of DIC. It is necessary to use large volumes of plasma in order to correct coagulation defects associated with a prolonged APTT or PT (greater than 1.5 times the normal value) or decreased fibrinogen level (less than 1.5 g/dl). An initial dose of 15 ml/kg of FFP is clinically recommended and usually administered. As the consequences of volume overload must be considered in this context, smaller volumes of prothrombin complex concentrate may be useful in this setting. As specific deficiencies in fibrinogen associated with the massive bleeding type of DIC can be corrected with the administration of purified fibrinogen concentrates or cryoprecipitate, three of the guidelines recommended these treatments (Table 3). The response to blood component therapy should be monitored both clinically and with repeated assessments of the platelet count and coagulation parameters following the administration of these components. The efficacy and safety of recombinant factor VIIa in DIC patients with life-threatening bleeding are unknown, and this treatment should be used with caution or as part of a clinical trial.

#### Heparin

Although the administration of anticoagulant treatment is a rational approach based on the notion that DIC is characterized by extensive activation of coagulation, there are several differences in the recommendations for the use of heparin in DIC patients between the four guidelines (Table 1) [3–6]. Therapeutic doses of heparin should be considered in cases of DIC in which thrombosis predominates. A small RCT showed that low molecular weight heparin (LMWH) is superior to unfractionated heparin (UFH) for treating DIC [45], suggesting that the use of LMWH is preferred to that of UFH in these cases. The level of inhibition achieved with LMWH is higher for activated coagulation factor Xa (Xa) than for thrombin. Patients with DIC are at high risk of VTE events, and the administration of VTE prophylaxis using UFH, LMWH, and/or mechanical methods has become the standard of care in patients with DIC [46, 47]. Although experimental studies have shown that heparin can at least partly inhibit the activation of coagulation in the setting of DIC [48], there are no RCTs demonstrating that the use of heparin in patients with DIC results in improvements in clinically relevant outcomes. A recent large trial of patients with severe sepsis showed a non-significant benefit of low-dose heparin on the 28-day mortality and underscored the importance of not discontinuing heparin treatment in patients with DIC and abnormal coagulation parameters [29]. Meanwhile, the 28-day mortality is lower in placebo groups treated with heparin than in placebo groups without heparin according to subclass analyses [49] of RCT of severe sepsis [37, 38, 42]. Although it is not easy to quickly measure the AT level in all hospitals in order to decide whether to administer urgent heparin treatment, measuring this parameter is useful for achieving the full efficacy of heparin. The administration of heparin is not recommended in patients with bleeding or massive bleeding type of DIC due to the increased risk of bleeding, although it is recommended in those with the non-symptomatic type of DIC in order to prevent the onset of deep vein thrombosis (DVT) (Table 3).

#### Anti-Xa agents

Both Fondaparinux® and Danaparoid sodium® activate AT specifically to inhibit Xa. Treatment with Fondaparinux® is recommended for the prophylaxis of DVT after orthopedic surgery; however, there is little evidence to support its use in critically ill patients and those with other type of DIC. Danaparoid sodium® is used to treat DIC in Japan, although no RCTs have shown any reductions in mortality or the rate of resolution of DIC. There is significant evidence for the use of these drugs as prophylaxis for DVT [50, 51]; however, there is little evidence for the use of these agents in patients with DIC, and they are not recommended in those with the bleeding or massive bleeding type of DIC (Table 3). These drugs are also not recommended in patients with renal failure.

#### Synthetic protease inhibitors

Synthetic protease inhibitors, such as Gabexate mesilate® and nafamostat®, exhibit multiple-functions, including antagonistic effects on the kinin/kallikrein system, fibrinolysis, complement system, and coagulation system. Gabexate mesilate® and nafamostat® have been frequently used and evaluated in Japan [13, 52, 53]; however, there are no RCTs showing any reductions in mortality or improvements in the rate of resolution of DIC. As these drugs have mild anticoagulant and antifibrinolytic effects, they are often used in patients with the bleeding, massive bleeding, and non-symptomatic types of DIC (Table 3).

#### Natural protease inhibitor

The use of agents capable of restoring dysfunctional anticoagulant pathways in patients with DIC has been studied extensively. Although there are many RCTs of clinically ill patients, almost all RCTs have been carried out in patients with sepsis, with few RCTs of patients with DIC, suggesting that BCSH and SISET determined their recommendations for DIC treatment based on studies of sepsis, not DIC.

**Figure 2:**
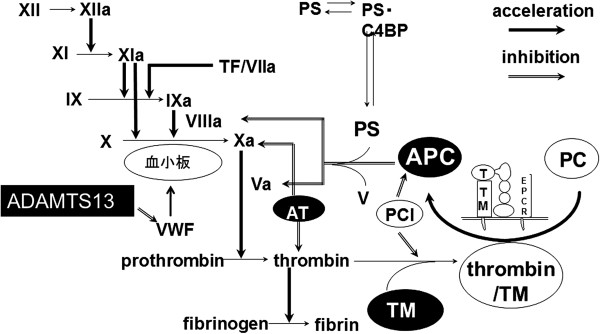
**Regulation of the coagulation system.**

AT and the heparin/heparinoid complex primarily inhibits Xa and thrombin, while the APC/TM system inhibits thrombin, FVa, and FVIIIa (Figure 2). Each of the four guidelines [3–6] provides different recommendations regarding the use of anticoagulant factor concentrates (Table 1). A large-scale multicenter RCT directly assessing the effects of AT concentrate on mortality in patients with severe sepsis showed no significant reductions in those treated with AT concentrate [37]. Interestingly, the subgroup of patients with DIC and who did not receive heparin showed a remarkable survival benefit [54]; however, this finding requires prospective validation. In one prospective multicenter survey, the efficacy of AT was higher in the 3,000 units/day group than in the 1,500 units/day group [55].

The clinical efficacy of recombinant human activated protein C (rhAPC) in patients with severe sepsis was demonstrated in a large RCT [38], although a prospective trial of septic patients with relatively low disease severity did not show any benefits of rhAPC therapy [39]. The withdrawal of rhAPC from sepsis treatment regimens was proposed after an RCT of septic shock failed to show any benefits [40]. Meanwhile, treatment with plasma-derived APC improved outcomes in a small RCT [56] in Japan; however, the drug is not approved for the treatment of DIC. There are no useful RCTs of the administration of protein C concentrate to treat sepsis or DIC.

One RCT comparing treatment with rhTM with that of UFH [43] showed that rhTM therapy significantly increased the rate of resolution of DIC, although mortality was not significantly decreased. In another study of DIC, treatment with rhTM relatively reduced mortality and significantly reduced the severity of organ failure compared to a placebo [57]. Another RCT of severe sepsis showed that the administration of rhTM tended to improve mortality [41].

The administration of AT, rhTM, or APC may be considered in DIC patients. Further prospective evidence from RCTs confirming a benefit is required [6]. Treatment with AT and rhTM is recommended in patients with the organ failure type of DIC (Table 3).

#### Antifibrinolytic treatment

Antifibrinolytic agents are effective in treating bleeding, although the use of these drugs in patients with the organ failure or non-symptomatic type of DIC is generally not recommended [58]. An exception may be made in those with the bleeding or major bleeding type of DIC. The four guidelines [3–6] exhibit some differences in these recommendations (Table 1). One study of APL demonstrated a beneficial effect of antifibrinolytic agents in this situation [59]; however, cases complicated with severe thrombosis due to the combined use of ATRA and tranexamic acid have been documented [60]. A recent RCT [61] showed that treatment with tranexamic acid significantly reduces the mortality of patients with trauma. The administration of antifibrinolytic agents in these cases must occur in the early period of management before the levels of PAI-1 and other endogenous antifibrinolytics become elevated.

## Conclusions

In conclusion, DIC is categorized into bleeding, organ failure, massive bleeding, and non-symptomatic types. The diagnosis and treatment of DIC should be carried out in accordance with the type of DIC based on the four guidelines on DIC.

## Abbreviations

ADAMTS13: a disintegrin-like and metalloproteinase with thrombospondin type 1 motifs 13
APL: acute promyelocytic leukemia
APTT: activated partial thromboplastin time
AT: antithrombin
ATRA: all-trans retinoic acid
BCSH: British Committee for Standards in Haematology
DIC: disseminated intravascular coagulation
FDP: fibrinogen and fibrin degradation products
FFP: fresh frozen plasma
FRMs: fibrin-related markers
HMGB-1: high mobility group box 1
ISTH: International Society of Thrombosis and Haemostasis
JAAM: Japanese Association of Acute Medicine
JMHLW: Japanese Ministry Health, Labour and Welfare
JSTH: Japanese Society of Thrombosis and Hemostasis
LMWH: low molecular weight heparin
LPS: lipopolysaccharide
NETs: neutrophil extracellular traps
PAI-I: plasminogen activator inhibitor I
PC: platelet concentrate
PT: prothrombin time
RCT: randomized controlled trial
rh: recombinant human activated protein C
SF: soluble fibrin
SISET: Italian Society for Thrombosis and Haemostasis
SSC: Scientific and Standardization Committee
TFPI: tissue factor pathway inhibitor
TM: thrombomodulin
UFH: unfractionated heparin
VTE: venous thromboembolism.
